# Effects of food hydrocolloids on quality attributes of extruded red Jasmine rice noodle

**DOI:** 10.7717/peerj.10235

**Published:** 2020-11-04

**Authors:** Supaluck Kraithong, Saroat Rawdkuen

**Affiliations:** Unit of Innovative Food Packaging and Biomaterials, School of Agro-Industry, Mae Fah Luang University, Muang, Chiang Rai, Thailand

**Keywords:** Extruded noodle, Quality characteristics, Hydrocolloids, Red Jasmine rice, Hom Mali Dang

## Abstract

The aim of this study was to examine the quality characteristics of extruded red Jasmine rice flour (RJF) noodle that had been prepared with hydrocolloids, namely guar gum (GG), carboxymethyl cellulose (CMC), and xanthan gum (XG) at the concentrations of 0.0 (control sample), 0.2, and 0.4% (w/w), respectively. The use of hydrocolloids had no effect on total phenolic contents, antioxidant properties (DPPH, ABTS, and FRAP), color, and X-ray diffraction patterns (*p* > 0.05). In contrast, the hydrocolloids tended to increase the expansion ration of the noodle. GG and CMC improved cooking, textural, and sensory properties. Ultimately, 0.2%-GG showed the lowest cooking loss (5.07%) when compared with others. Moreover, it also provided the noodle with better textural properties such as tensile strength, extensibility, hardness, cohesiveness, and chewiness (*p* < 0.05). For these reasons, the highest acceptability (6.75) for the noodle was achieved with GG02. XG resulted in lower overall acceptability (5.05), particularly the 0.4%-XG recipe (*p* < 0.05). Thus, usage of 0.2%-GG was the best option for improving the qualities of extruded RJF noodle. XG was deemed ineffective for improving the noodle.

## Introduction

Thai red Jasmine rice (Hom Mali Dang) is a premium food product from Thailand. The cooked grain has a unique fragrance and softness, and exports of this rice variety increased from 123,740 metric tons in January of 2018 to 171,811 metric tons in December of the same year (Thai rice exporters association, 2018). Moreover, Thai red Jasmine rice has also been used as raw material for producing health foods, food supplements, and also nutraceutical products. This is due to the numerous bioactive compounds (i.e., anthocyanin, phenolic acids) that are able to prevent or retard oxidative reactions, making it well-known as a natural antioxidant source (Abdel-Aal, Young & Rabalski, 2006). Furthermore, the red rice also has low blood glucose levels (glycemic index; GI) that could slow the development of some chronic metabolic diseases (Sumczynski et al., 2016).

In Thailand, the red rice has been applied for producing one of the most famous traditional Asian foods, rice noodle. That notwithstanding, there have been few scientific reports about this particular strain of noodle. Traditionally, rice noodle processing consists of many steps. Firstly, the whole or broken rice grains will be soaked and then ground to obtain rice slurry. After that, the slurry is steamed for around 3–5 min. Then, the noodle sheet will be obtained and further dried with many steps of drying to decrease moisture content (Wu et al., 2015). It takes approximately more than 20 h to produce one batch. For this reason, traditional rice noodle manufacturing has been regarded as laborious and time consuming. Moreover, the many steps it takes to produce rice noodles also results in inconsistencies in quality, and so also in consumer satisfaction (Tan, Li & Tan, 2009). These are crucial issues for rice noodle manufacturers. Hence, an extrusion technique is hereby submitted for rice noodle production to overcome these disadvantages. For rice noodle extrusion, rice flour will be used to adjust moisture content, and then it will be subjected to an extruder machine. Afterward, rice noodle strands will be obtained from the die and put through a drying process. Fewer steps and a continuous extrusion process may give higher efficiency for controlling product quality, and thereby result in increasing acceptability and lessening waste. It is also expected that this shorter process will save energy and time (Charutigon et al., 2008).

It is typical for rice noodles prepared by rice flour with low amylose content (<20%), such as red Jasmine rice flour, to have poor qualities like texture, cooking loss, and other sensory properties. This is due to less development of three dimensional networks of the amylose (Wang et al., 2016). Thus, hydrocolloids have been used for fixing this problem because of their ability to immobilize water molecules in the polymer chains. In traditional rice noodle processing, hydrocolloids such as guar gum (GG) obtained from *Cyamopsistetra gonoloba* seed, carboxymethyl cellulose (CMC) gained from derivative of cellulose, and xanthan gum (XG) developed from *Xanthomonas campestris*, have all been reported to improve noodle qualities by reducing solid loss via interactions or bonding of their polymer chains. Cai et al. (2016) confirmed that XG increased tensile strength, hardness, and chewiness of gluten-free noodles from glutinous rice flour. Loubes, Flores & Tolaba (2016) found that 0.7%-GG gave the highest strain at break (17.9%) and the lowest cooking loss (13%) for rice noodle products. Suwannaporn & Wiwattanawanich (2011) also reported that 1.5%-CMC reduced cooking loss in wheat-rice noodle, and also improved tension force and the breaking distance of the noodle.

Undoubtedly, hydrocolloids are surely able to improve quality attributes of rice noodle prepared using the conventional process. In the extrusion process, the ability to improve food product qualities of hydrocolloids can be altered by temperature and pressure generating (Kaur et al., 2015). Besides, the interactions among the polymer chains found to be somehow disturbed by the extrusion conditions (Maskan & Altan, 2016). In other words, the hydrocolloids that are able to improve the quality attributes of rice noodle prepared from the traditional method may not show the same capability in the extrusion process; hydrocolloids that improved the qualities of noodle prepared using the conventional process may not improve extruded rice noodle properties. Thus, the qualities of noodle made from rice flour with hydrocolloids by extrusion method may be different from that of the traditional method. Therefore, this work had examined the quality attributes of extruded red Jasmine rice noodle prepared with different types and levels of hydrocolloids. To find out the best hydrocolloid, with a proper concentration, that effectively improves rice noodle properties under extrusion conditions. The results from this work can be as supporting information for applying hydrocolloids into other extruded products.

## Materials & Methods

### Raw materials

Red Jasmine rice (Hom Mali Dang) grains were purchased from Siam organic food products Co., Ltd. (Bangkok, Thailand). The rice was grown in Thailand and harvested in 2017.

For preparing the red Jasmine rice flour (RJF), the rice kernels were crushed with a hammer mill (CMC-20, Thailand). Then, the rice powder was sieved with a 60 mesh sieve.

GG and XG were bought from Wendt-chemie GmbH (Hamburg, Germany). Their viscosities were 3870 mPa.s and 1722 mPa.s at 1% solution, respectively. CMC (FVH6-A) was bought from Changshu Wealthy Science and Technology Co., Ltd. (Changshu, China). Its viscosity was 2,400 to 2,600 mPa.s at 2% solution. In this study, all hydrocolloids used were food grade.

### Extrusion processing

The red Jasmine rice flour (RJF) was mixed with the different levels (0.2% and 0.4% w/w) of the hydrocolloids (GG, CMC, and XG); where RJF mixed with 0.2% GG = GG02, RJF mixed with 0.4% = GG04, RJF mixed with 0.2% CMC = CMC02, RJF mixed with 0.4% CMC = CMC04, RJF mixed with 0.2% XG = XG02, and RJF mixed with 0.4% XG = XG04. The rice flour without adding hydrocolloids was used as a control.

Before the extrusion process, the mixtures were adjusted for moisture content to 35% following the Eqs. (1) and (2), and then kept at 4 °C for 12 h to reach equilibrium (Zasypkin & Lee, 1998).

(1) \begin{eqnarray*}{M}_{r} = \frac{100\times M\times (100-\mathrm{W})}{100 \times (100-{\mathrm{W}}_{r})} \end{eqnarray*}

(2) \begin{eqnarray*} {\mathrm{M}}_{\text{water} }=M-{M}_{r}\end{eqnarray*}

M_r_ = Mass of RJF at an initial moisture content; M = Mass of RJF at 35% moisture content, which was needed for the extrusion process; W = Final moisture content (35%);

W_r_ = RJF moisture content; M_water_ = Water needed.

After moisture adjustment, the mixtures were subjected to a single screw extruder (Brabender, Model DO-CORDER C3, Germany). The extruder screw was divided into 3 zones with fixed temperatures at 70:70:80 °C. The feed rate and screw speed were set at 30 and 200 rpm, respectively. The width and length of a rectangle extruder die were one mm and 3.5 mm, respectively. The screw length per diameter (L/D) was set at 23.68:1 mm. An extrudate was cut into strips and dried at 40 °C (1 h), reducing moisture content to 12%. Then, the extruded noodle was kept in a vacuum bag.

### Determination of total phenolic content (TPC)

The extruded rice noodles were ground into powder by using a blender (HR2001, Philips, China) and extracted based on the method described in Abdel-Aal, Young & Rabalski (2006). The samples (1.0 g) were added with 10 mL of 85% methanol, and then stirred at 25 °C for 30 min. The suspensions were centrifuged (AVANTI j-30I, Beckman, Germany) at 2500 g for 10 min. Then, supernatants were stored in the dark at 4 °C.

Determination of TPC was conducted based on the procedure of Chan et al. (2012). The extracted solutions (0.1 mL) were added with five mL of 10% (v/v) Folin-Ciocalteu reagent and 0.4 mL of 7.5% (w/v) sodium bicarbonate solution. Then, the solutions were incubated in the dark for 1 h. Absorbance at 765 nm was then measured with a microplate reader (Multiskan Go, Thermo Scientific, Finland). The result was expressed as mg gallic acid equivalents (GAE)/100 g DW sample.

### Determination of antioxidant activities

Measurement of DPPH radical-scavenging activity was conducted according to the method described in Chan et al. (2012). The extracted solutions (50 µL) were added with 0.1 mM DPPH methanolic solution (1,950 µL). After that, they were kept in the dark for 1 h before measuring absorbance at 540 nm by a microplate reader. The DPPH was estimated against the Trolox standard curve and expressed as µmol Trolox equivalent/100 g DW sample.

ABTS radical scavenging activity was estimated as described by Chatatikun & Chiabchalard (2013). The extracted solutions (50 µL) were mixed with 950 µL of ABTS working solution. The solutions were kept in the dark for 6 min followed by absorbance measurement at 734 nm with a microplate reader. The ABTS was calculated as µmol Trolox equivalent/100 g DW sample.

Measurment of FRAP was executed according to the method of Corral-Aguayo et al (2008). The extracted solutions (20 µL) were added with 180 µL of FRAP reagent. The solutions were then incubated for 30 min in the dark before measuring absorbance at 630 nm by a microplate reader. FRAP was expressed as µmol ferrous sulfate equivalent (Fe (II))/100 g DW sample.

### Color attributes

Measurement of color (CIELAB) for the extruded noodle samples was conducted by using a colorimeter (Miniscan EZ, USA). The machine was standardized with a white calibration tile. The samples were measured for their values of lightness (L*), redness (a*), and yellowness (b*).

For Munsell color system, the hue angle (h*; basic color) and chroma (C*; color intensity or saturation) were calculated using the following equations:

(3) \begin{eqnarray*}{\mathrm{h}}^{\ast }={\text{Tan}}^{-1} \left( \frac{{b}^{\ast }}{{a}^{\ast }} \right) \end{eqnarray*}

(4) \begin{eqnarray*}{\mathrm{C}}^{\ast }=\sqrt{{a}^{\ast 2}+{b}^{\ast 2}}\end{eqnarray*}

The total color difference (ΔE) was estimated in relation to control sample using the following equation:

(5) \begin{eqnarray*}\Delta E=\sqrt{({L}_{}^{\ast }b+{L}_{}^{\ast }a)^{2}+({a}_{}^{\ast }b+{a}_{}^{\ast }a)^{2}+({b}_{}^{\ast }b+{b}_{}^{\ast }a)^{2}}\end{eqnarray*}

Where L* _a_, a* _a_ and b* _a_ values are from the control while L* _b_, a* _b_ and b* _b_ are values of the extruded noodle with hydrocolloids.

### X-ray diffraction pattern (XRD) and crystallinity

XRD pattern and crystallinity were investigated with an X-ray diffractometer (X’Pert Pro MPD, PANalytical, Japan) based on of the method described in Thumrongchote et al. (2012). The (ground) extruded noodle samples were added into a sample holder ring and pressed with a powder press block. Then, the samples were removed and placed in an X-ray diffractometer, which was set at the following conditions: 40 kV, 30 mA using K *α* X-rays for the 2 *θ* range of 4-−40° with a resolution of 0.05° step size. The percentage of crystallinity was calculated by the following equation:

(6) \begin{eqnarray*}\mathrm{Percentage~ of~ crystallinity} (\text{%})= \left( \frac{\mathrm{Total} \mathrm{crystalline~ peak~ area}}{\mathrm{Crystalline~ and~ amorphous}\mathrm{peak~ areas}} \right) \times 100.\end{eqnarray*}

### Expansion ratio

Measurement of expansion ratio was conducted by estimating the ratio of noodle diameter to die orifice diameter (Baek & Lee, 2014). The diameter of the extruded noodle samples (with an average of 15 random measurements) was measured using a dial caliper (Smiec, China).

### Cooking properties

Cooking time was investigated following the procedure described in Wu et al. (2015). The extruded noodle (5 g) was cut into strands six cm in length. After that, they were cooked with 200 mL of boiling distilled water. This property was observed when the noodle core disappeared. Noodle core observation was done by squashing the rice noodle strand between two glass plates every 30 s.

Determination of cooking loss and rehydration was performed under the same conditions as described above for cooking time. Cooked rice noodle strands were rinsed with 50 mL of distilled water. The water used for cooking and rinsing was kept and then dried at 105 °C until a constant weight was reached. The percentage of cooking loss was estimated by the following equation:

(7) \begin{eqnarray*}\mathrm{Cooking~ loss} (\text{%}) = \left( \frac{\mathrm{Weight~ of~ dry~ matter~ in~ cooking~ water} (g)}{\mathrm{Weight~ of~ dry}\mathrm{noodle} (g)} \right) \times 100.\end{eqnarray*}

The cooked noodle strands were taken out. Afterwards, the excess water was removed from the noodle surfaces by using a paper towel (Von Loesecke, 1954). The rehydration value was calculated by using the following equation:

(8) \begin{eqnarray*}\text{Rehydration} (\text{%}) = \left( \frac{\text{Weight of cooked noodle} (g)-\text{weight of uncooked noodle} (g)}{\text{Weight of uncooked noodle} (g)} \right) \nonumber\\\displaystyle \times 100.\end{eqnarray*}

### Texture properties

Determination of texture properties was done by using a texture analyzer (model TA. XT. Plus, Stable MicroSystems Ltd., England) based on the method of Ye & Sui (2016). The extruded noodle was cooked for the optimal cooking duration. For compression testing, the noodle strands were compressed with a hemispherical probe (P/0.5HS) with a test speed of 2.0 mm/s and with 30% strain. The texture properties tested included hardness (g), adhesiveness (g sec), cohesiveness, gumminess (g), springiness, and chewiness (gmm). For the tension test, values of tensile strength (g) and extensibility (mm) were determined by using a pair of spaghetti/noodles tensile grips with a cross head velocity of 3.0 mm/s.

### Statistical analysis

All experimental measurements were conducted in triplicate. However, there were six replications for texture analysis. Analysis of variance (ANOVA) was conducted, and means comparison was achieved by Duncan’s Multiple Range Tests (DMRT). The significance of difference was determined to be *p* < 0.05. The analysis was completed with an SPSS package (SPSS 17.0 for window, SPSS Inc, Chicago, 180 IL).

## Results

### TPC and antioxidant activities

**Figure 1:**
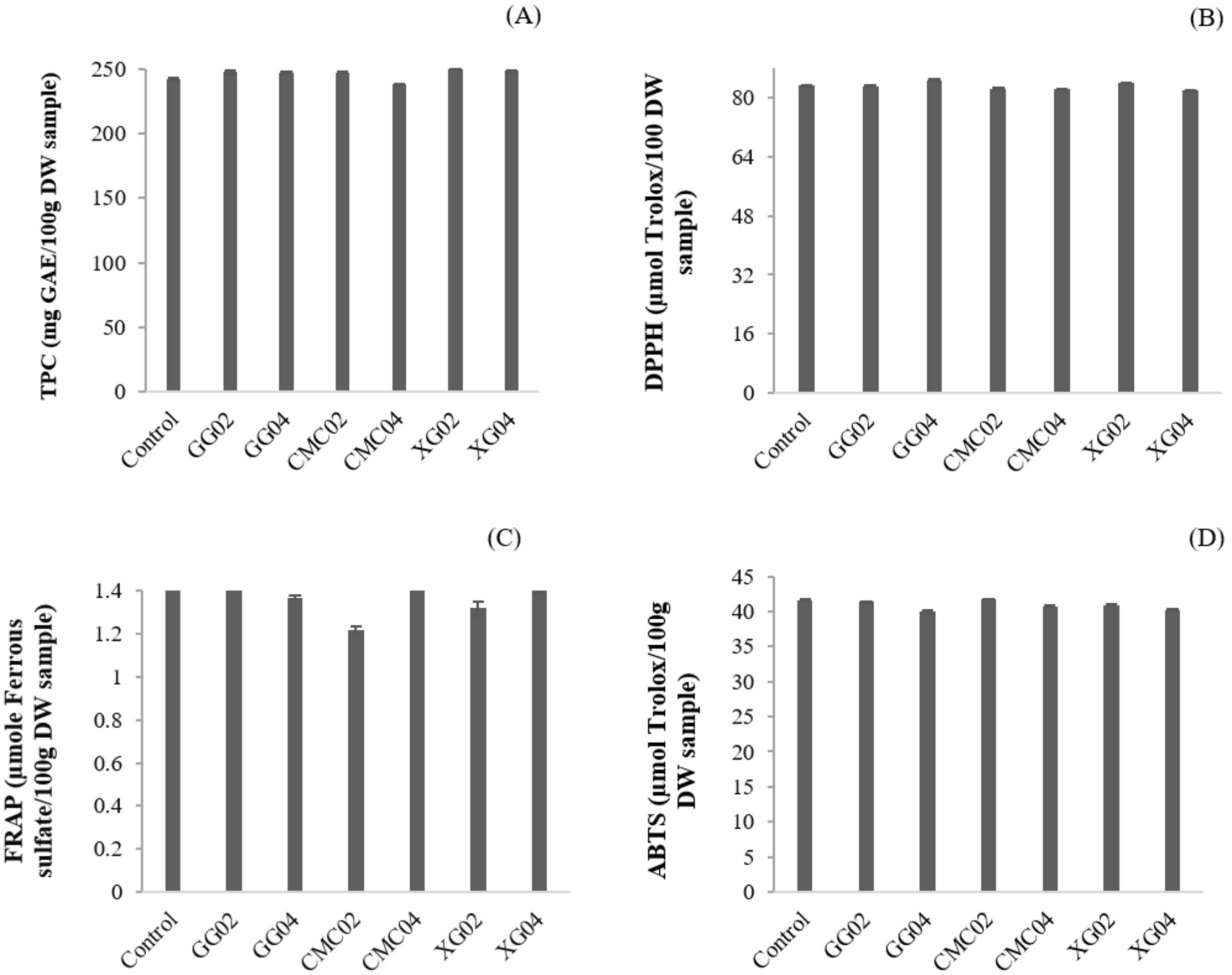
Total phenolic compounds and antioxidant activities of extruded red Jasmine rice flour noodle powders. (A) Total phenolic content, (B) DDPH radical-scavenging activity, (C) Ferric reducing antioxidant power, (D) ABTS radical-scavenging activity.

In this study, there were no effects by GG, CMC, and XG observed on TPC; nor were there any antioxidant activities observed in the extruded noodle (*p* > 0.05) due to a low concentration used. The TPC value was in the range of 230.07–250.18 mg GAE/100 g DW sample (Fig. 1). Antioxidant properties, namely DPPH, ABTS, and FRAP, were found in the range of 81.16–84.59 µmol Trolox/100 g DW sample, 39.99–41.59 µmol Trolox/100 g DW sample, and 1.22–1.47 µmol Fe(II)/100 g DW sample (Fig. 1), respectively.

### Color attributes

The color attributes (CIELAB) of the extruded noodle were not affected by usage of the hydrocolloids (*p* > 0.05). Values of L*, a*, and b* in the extruded noodle were in the range of 21.80–23.40, 5.11–6.30, and 5.40–6.70 (Table 1), respectively. For Munsell color system, the h* values of the noodle samples were 42.40–52.77 (Table 1). This indicated that the samples still maintained their redness. This value can be used to indicate the color from red to blue (Saricoban & Yilmaz, 2010); h* = 0°, 90°, 180°, and 270° indicating redness, yellowness, greenness, and blueness, respectively. However, the use of hydrocolloids found to significantly decrease H° of the noodle.

### XRD patterns and crystallinity

**Figure 2:**
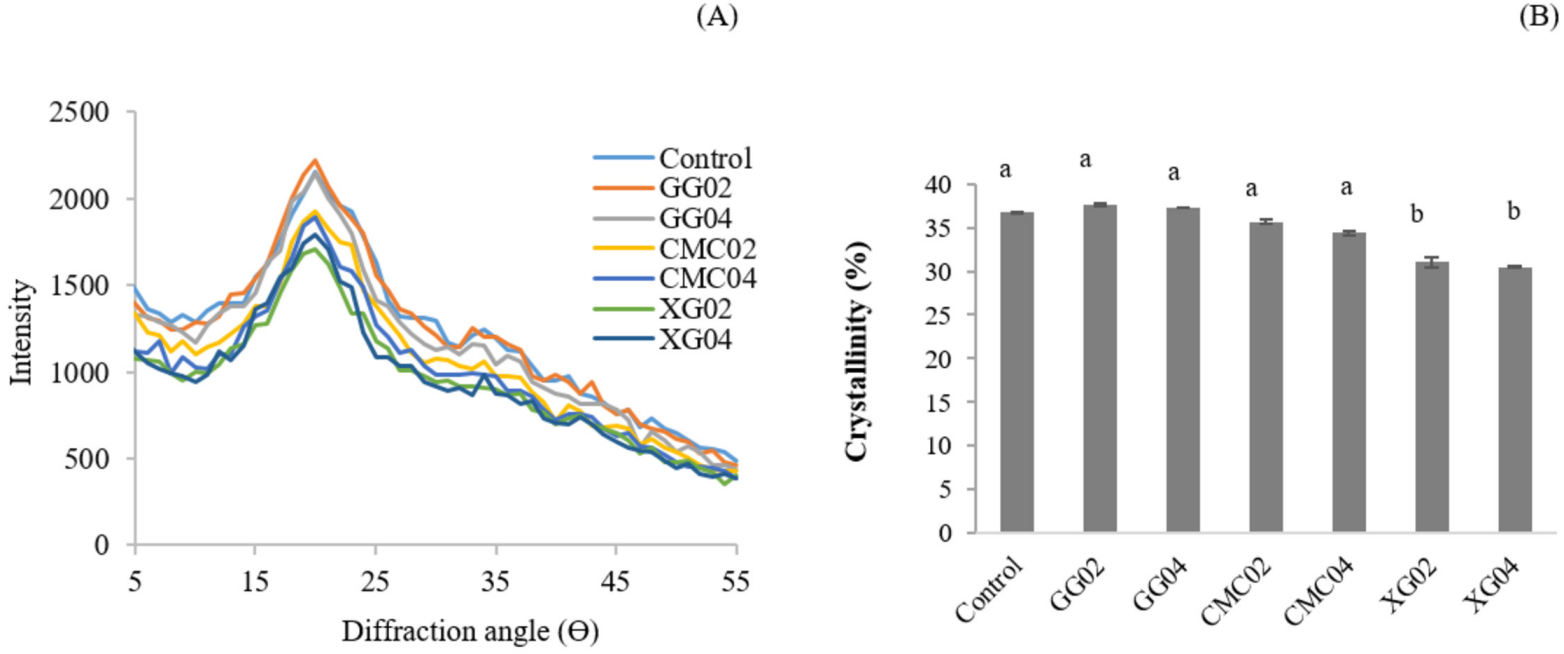
X-ray diffractograms (A) and crystallinity (B) of extruded red Jasmine rice flour noodle powders.

All extruded noodle samples demonstrated a V-type pattern, which was not affected by using hydrocolloids. They exhibited a peak at 2 Θ of 17° (Fig. 2) because of the amylose-lipid complex formation. This result shows that using hydrocolloids has no effect on the XRD pattern of the noodle. On the other hand, the results showed that the lower intensity was provided by XG. The noodle sample XG04 had the lowest crystallinity (30.50%) (Fig. 2). While the use of GG and CMC had no significant effect on this property. The higher intensity was found in control, GG02, as well as GG04 which refers to the more stability of crystallinity in those samples.

**Table 1:** Color attributes and cooking properties of the extruded red Jasmine rice flour noodles with hydrocolloids.

**Sample**		**Color parameters**							**Cooking properties**			
		L*	a*	b*	Hue angle	Chroma	ΔE	Cooking time (min)		Cooking loss (%)	Rehydration (%)	
Control 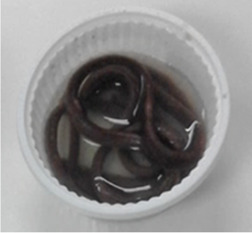		21.85 ± 1.48^ab^	5.11 ± 0.69^ab^	6.70 ± 0.28^a^	52.77 ± 3.42^a^	8.43 ± 0.55^a^	–	8.47 ± 0.05^b^		6.01 ± 0.41^b^	103.97 ± 0.5^c^	
GG02 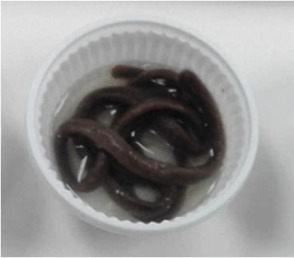		23.10 ± 0.57^a^	6.30 ± 0.21^a^	5.55 ± 0.21^ab^	41.39 ± 0.13^c^	8.39 ± 0.30^a^	2.26 ± 0.91^a^	9.10 ± 0.11^a^		5.07 ± 0.37^c^	160.14 ± 2.01^ab^	
GG04 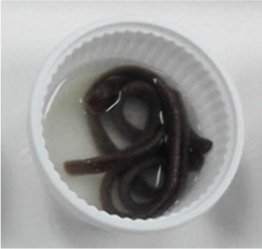		21.80 ± 0.42^ab^	5.50 ± 0.47^a^	6.30 ± 0.57^a^	48.86 ± 2.96^ab^	8.37 ± 0.60^a^	1.45 ± 0.55^b^	9.03 ± 0.07^a^		5.15 ± 0.21^c^	163.75 ± 1.03^b^	
CMC02 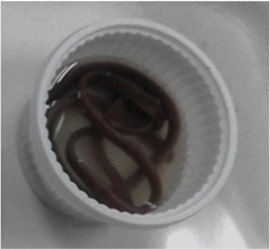		22.90 ± 0.85^a^	6.20 ± 0.31^a^	6.20 ± 0.15^a^	45.02 ± 0.76^bc^	8.77 ± 0.33^a^	2.41 ± 0.80^a^	8.43 ± 0.03^b^		5.32 ± 0.23^c^	163.37 ± 1.75^b^	
CMC04 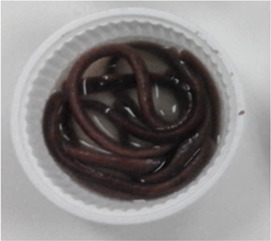		23.00 ± 0.19^a^	5.95 ± 0.07^ab^	5.60 ± 0.11^ab^	43.26 ± 0.24^c^	8.17 ± 0.12^a^	2.18 ± 0.90^a^	8.53 ± 0.10^b^		5.27 ± 0.97^c^	166.59 ± 1.31^b^	
XG02 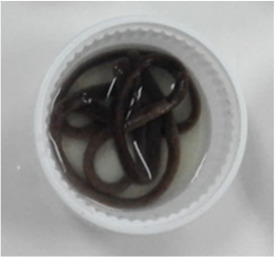		22.30 ± 0.33^a^	5.89 ± 0.13^ab^	5.40 ± 0.71^ab^	42.40 ± 3.42^c^	8.00 ± 0.54^a^	2.24 ± 0.52^a^	8.27 ± 0.09^c^		7.25 ± 0.43^ab^	172.22 ± 1.79^a^	
XG04 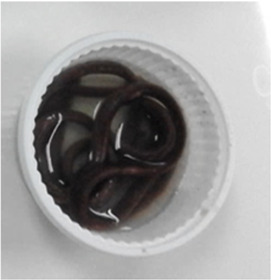		23.40 ± 0.41^a^	5.71 ± 0.19^ab^	5.75 ± 0.93^a^	44.99 ± 4.38^bc^	8.12 ± 0.72^a^	2.23 ± 1.43^a^	8.27 ± 0.07^c^		7.97 ± 0.81^a^	173.64 ± 2.37^a^	

**Notes.**

All values are means ± standard deviation (*n* = 3).

a–c Means with the same superscript letters within a column are not significantly different at *p* < 0.05 level. Red Jasmine rice flour (Control); red Jasmine rice flour + 0.2% guar gum (GG02); red Jasmine rice flour + 0.4% guar gum (GG04); red Jasmine rice flour + 0.2% CMC (CMC02); red Jasmine rice flour + 0.4% CMC (CMC04); red Jasmine rice flour + 0.2% xanthan gum (XG02); red Jasmine rice flour + 0.4% xanthan gum (XG04).

### Expansion ratio

**Figure 3:**
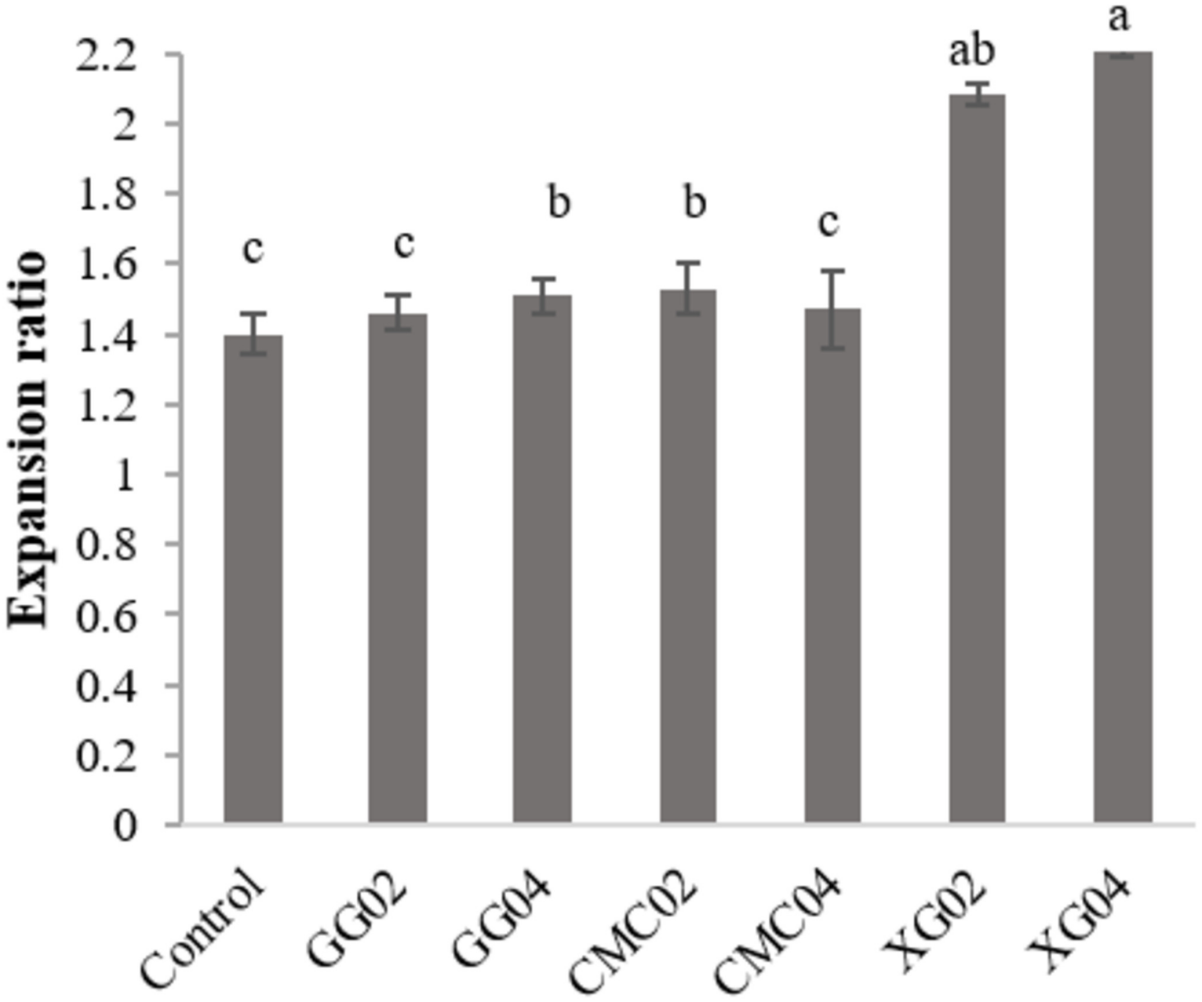
Expansion ratio of extruded red Jasmine rice flour noodles.

This study found that the use of all hydrocolloids; GG, CMC, and XG significantly increased expansion ratio for the extruded noodle (*p* < 0.05). The expansion ratio of the extruded noodle was increased by nearly 8%, 10%, and 37% (Fig. 3) when GG, CMC, and XG were used (*p* < 0.05), respectively. The highest expansion ratio of the extruded noodle provided by the addition of 0.4% XG (*p* < 0.05).

### Cooking properties

Using 0.2% GG gave the extruded rice noodle the longest cooking time (9.10 min) (Table 1) (*p* < 0.05). Conversely, XG reduced this property (*p* < 0.05). Thus, the shortest cooking time (8.27 min) was found when using 0.2% and 0.4% XG (*p* < 0.05). Whereas, the use of CMC had no effect on cooking time as indicated with no change in crystallinity (*p* < 0.05).

The highly branched hydrocolloid which is XG tended to increase the cooking loss of the extruded rice noodle. The highest cooking loss (7.97%) was found in the noodle sample XG04 (Table 1). In contrast, the use of GG and CMC showed the opposite result; the two hydrocolloids significantly reduced the solid loss of the extruded rice noodle.

Percentage of rehydration in the extruded rice noodle was increased by about 36%, 38%, and 40% by using GG, CMC, and XG (Table 1), respectively (*p* < 0.05). The use of the highly branched hydrocolloid (XG) given the greatest rehydration to the extruded noodle compared to other polymers (*p* < 0.05).

### Textural properties

The results showed that GG and CMC improved all textural properties of extruded rice noodle. They reduced the cooking loss value by the interactions of their polymer chains. Ultimately, using 0.2%-GG gave the extruded noodle the highest values of some textural properties such as tensile strength (80.95 g), extensibility (27.42 mm), hardness (1,798 g), cohesiveness (0.65), gumminess (1,174.68 g), and chewiness (987.00 gmm) (Table 2). However, tensile strength, extensibility, and hardness of the extruded noodle found to be decreased by the addition of XG (*p* < 0.05).

**Table 2:** Textural properties of the extruded red Jasmine rice flour noodles with hydrocolloids.

**Sample**	**Textural properties**						
	Tensile strength (g)	Extensibility (mm)	Hardness (g)	Adhesiveness (gsec)	Cohesiveness	Gumminess (g)	Chewiness (gmm)
Control	65.45 ± 5.32^b^	16.10 ± 1.93^b^	1119.57 ± 38.16^bc^	−25.42 ± 0.97^c^	0.46 ± 0.02^c^	510.35 ± 33.83^c^	408.01 ± 91.27^c^
GG02	80.95 ± 9.68^a^	27.42 ± 3.11^a^	1798.58 ± 46.71^a^	−40.70 ± 1.21^a^	0.65 ± 0.11^a^	1174.68 ± 191.61^a^	987.00 ± 101.93^a^
GG04	78.23 ± 8.59^a^	27.41 ± 4.57^a^	1766.10 ± 70.23^a^	−41.27 ± 1.11^a^	0.61 ± 0.05^ab^	1071.85 ± 85.43^a^	858.65 ± 91.11^b^
CMC02	77.80 ± 6.31^a^	26.14 ± 5.93^a^	1782.72 ± 89.23^a^	−37.61 ± 1.67^b^	0.59 ± 0.05^b^	1043.72 ± 110.81^ab^	872.22 ± 77.33^b^
CMC04	80.61 ± 10.63^a^	27.19 ± 6.71^a^	1738.97 ± 88.16^a^	−39.74 ± 0.91^b^	0.57 ± 0.07^ab^	993.79 ± 139.41^b^	803.31 ± 103.27^b^
XG02	54.31 ± 11.65^c^	17.75 ± 5.05^b^	1074.21 ± 66.61^c^	−42.06 ± 1.77^a^	0.50 ± 0.03^bc^	588.81 ± 46.27^c^	458.61 ± 11.03^c^
XG04	53.19 ± 4.77^c^	17.45 ± 6.27^b^	1143.96 ± 56.57^b^	−42.16 ± 0.71^a^	0.53 ± 0.09^c^	517.88 ± 85.51^c^	447.60 ± 75.18^c^

**Notes.**

All values are means ± standard deviation (*n* = 6).

a-c Means with the same superscript letters within a column are not significantly different at *p* < 0.05 level.

* Springiness was not different among the samples.

Red Jasmine rice flour (Control); red Jasmine rice flour + 0.2% guar gum (GG02); red Jasmine rice flour + 0.4% guar gum (GG04); red Jasmine rice flour + 0.2% CMC (CMC02); red Jasmine rice flour + 0.4% CMC (CMC04); red Jasmine rice flour + 0.2% xanthan gum (XG02); red Jasmine rice flour + 0.4% xanthan gum (XG04).

## Discussion

### TPC and antioxidant activities

Hydrocolloids have been reported to show an effect on antioxidant activities in food products. Saberi et al. (2017) reported that bonding between hydrocolloid hydroxyl groups and phenolic compounds can cause a decrease in TPC and antioxidant activity. Yi et al. (2015) also insisted that hydrocolloids are able to reduce the total antioxidant capacity of grape juice. However, the TPC and antioxidant activities of the extruded rice noodles are not different (*p* > 0.05) due to the small amount of the hydrocolloids but the values were lower than the noodle prepared by the conventional method. Kraithong, Lee & Rawdkuen (2018) found that TPC, DPPH, FRAP, and ABTS in red Jasmine rice noodle that was prepared by conventional method, were 529.19–540.96 mg GAE/100g DW sample, 171-178 µmol Trolox/100 g DW sample, 117-126 µmol Trolox/100 g DW sample, and 2.88–3.01 µmol Fe(II)/100 g DW sample, respectively. This could be caused by the destruction of rice flour pigments throughout the extrusion process, specifically due to pressure (over 200 rpm) and heat (over 80 °C) (Giles Jr, Wagner & Mount, 2013). Varzakas & Tzia (2015) also confirmed that extrusion temperature at 80 °C could destroy bioactive compounds and natural antioxidants in cereal and vegetable based food products. Thus, it can be concluded that the TPC and antioxidant activities of the rice noodle are highly influenced by the noodle process than the addition of hydrocolloids.

### Color attributes

In a previous study, L*, a*, and b* values of red Jasmine rice noodle, prepared by the conventional method, were 51-53, 12-14, and 15-16, respectively. Thus, the extrusion technique provides rice noodle with a darker color when compared to the traditional method. This is due to the Maillard reaction development. The reaction could occur during the extrusion process, when there is interaction between carbonyl groups of reducing sugars (e.g., glucose, maltose, fructose) and amino acids (mostly lysine and arginine), which is supported by high temperature (Varzakas & Tzia, 2015). Maskan & Altan (2016) also reported that temperature of extrusion conditions (over 80 °C) and low moisture content (lower than 40%) of raw materials resulted in a browning reaction in foods rich in starch. Under these conditions, the colors of the food products were made darker. Mesquita, Leonel & Mischan (2013) also found that extrusion temperature at 90−130 °C and a screw speed of 190-270 rpm contributed to the caramelization or browning of sugar (giving dark color) in an extruded snack from blending cassava starch and flaxseed flour.

The significant decrease of H° by adding hydrocolloids could be due to an increase in heat and thermal generating while hydrocolloids were added, destroyed the pigments are responsible for the redness (Baek & Lee, 2014). Whereas, the c* was not influenced by the addition of hydrocolloids (*p* > 0.05) indicated that the purity or saturation of the sample color is not different. The ΔE values of the extruded noodles with hydrocolloids were found from 1.45 to 2.26 (Table 1); the values are considerably low (Saricoban & Yilmaz, 2010). These indicated that the use of hydrocolloids showed only slightly effects on the total color; the color of the control sample and extruded noodles with hydrocolloids was nearly not different.

In general, anthocyanins which are a class of flavonoids are the major pigments are responsible to express plant colors from orange, red, purple to blue or black colors (Iwashina, 2015). The total anthocyanin contents in the red rice flour (a raw material) were found around 0.5–.07 cyanidin-3-glucoside equivalents, mg/L. The contents could not be detected in all noodle samples after the extrusion processing. Thus, it can be concluded that the extrusion process could destroy the main pigment which responsible for noodle redness. However, the detected redness in the samples could be responsible by other pigments such as carotenoids and betacyanins (Leong et al., 2018).

### XRD patterns and crystallinity

The formation of amylose-lipid complex was generally induced by heat and pressure during the extrusion process (Maskan & Altan, 2016). The lower intensity and crystallinity provided by XG indicates that the noodle samples with XG have lost their crystalline regions during the extrusion processing (Giles Jr, Wagner & Mount, 2013). This refers to a less ordered structure in the sample, causing a more fragile and unsteady structure for the noodle samples (Giles Jr, Wagner & Mount, 2013). The decreases in XRD peak intensity as well as crystallinity of the extruded noodle by adding XG may contribute to an undesired texture of the noodle. Detchewa et al. (2016) also insisted that a decrease in crystallinity resulted in an unstable structure (poor texture properties) of gluten-free rice spaghetti, weakening the spaghetti structure. Whereas, the higher intensity and crystallinity was found in control, GG02, as well as GG04 refers to the more stability of crystallinity in those samples.

### Expansion ratio

The increased expansion ratio of the extruded noodle by the additions of hydrocolloids could be because the polymers induce friction force or expand heat generation during the extrusion processing, resulting in an increasing degree of product puffing (Seth, Badwaik & Ganapathy, 2015). An increase in expansion ratio refers to the disruption or swelling of starch granules, contributing to the greater water absorption as well as more flexibility of the final product structure (Wang et al., 2016). Nevertheless, the extensive increase in expansion ratio can cause poor texture (unacceptable) for the noodle samples. This is because the noodle structure is extremely disrupted which caused brittle structure and less thermal resistance for the noodle during the cooking process (Kaur et al., 2015). The largest expansion (2.24) was found in the extruded noodle sample XG04 (*p* < 0.05). This could be in part due to a less ordered structure or lower crystallinity in the extruded noodle (*p* < 0.05). This refers to more starch granules collapsing and weaker construction of the noodle (Maskan & Altan, 2016), giving it a breakable structure.

### Cooking properties

The longest cooking time provide by 0.2% GG could be reinforced by less space in the noodle structure; moreover, that could also be supported by a denser or more ordered crystalline structure (*p* < 0.05). According to Detchewa et al. (2016), a more ordered crystalline structure could retard the penetration of water into the noodle core during cooking, leading to better textural qualities. Moreover, the application of the hydrocolloid shows to provide dense matrix to the noodle structure (Kaur et al., 2015). While the reduction of cooking time that provided by XG caused a more puffing texture in the noodle (more expansion ratio), representing the weakest structure and the most disrupted starch granules. Therefore, this could allow water to enter into its core more easily. Also, a less crystalline structure but a more amorphous region could support a short cooking time for a noodle product (Liu, Chaiwanichsiri & Laohasongkram, 2014).

The increased cooking loss of the extruded noodle was indicated by the higher expansion ratio and less ordered crystallinity (*p* < 0.05). Higher cooking loss is generally considered as an undesirable property for rice noodles because it indicates more solid components leaching out during cooking and less cooking tolerance (fragile structure), which produces more turbidity to cooking water (Wang et al., 2016). The reduced cooking loss given by GG and CMC caused by less expansion ratio when compared to that using XG. Reduced solid loss is also due to a greater ordered crystallinity (*p* < 0.05). In addition, hydrogen bonding among polymer chains of hydrocolloids could also reduce the amount of solid loss from the noodle structure during cooking due to the formation between the polysaccharide and starch molecules (Kaur et al., 2015). This finding is different from our previous report which showed that GG, CMC, and XG could significantly reduce cooking loss in the rice noodle which prepared using the traditional method. This is because friction which is generating throughout the extrusion process can weaken noodle structure. Thus, it can be inferred that the effects of hydrocolloids on rice noodle properties are also influenced by the production technique.

Hydrocolloids could increase the noodle rehydration because they have a good ability to attach or embrace water molecules by the hydroxyl groups of their polymer chains (Cai et al., 2016). Nevertheless, XG showed a better capacity for holding water because of the highly branched chains of XG and owning greater hydroxyl groups (Kaur et al., 2015). Moreover, less ordered structure of the samples indicated by the lower crystallinity and extensive expansion ratio could generate fast water absorption (Seth, Badwaik & Ganapathy, 2015). These also could increase the rate of rehydration. The increase of rehydration could contribute to an increasing stickiness in the rice noodle texture (Ye & Sui, 2016), which is an unrequired property for the rice noodle.

### Textural properties

The textural improvement provided by GG and CMC could be reinforced by the stronger structure of the rice noodle, contributed by the lowest solid loss and the greatest ordered crystallinity (*p* < 0.05). The texture improvement frequently refers to the better acceptability of the noodle product. Thus, the noodle sample had the highest acceptability. Kraithong, Lee & Rawdkuen (2018) insisted that the greatest increases in tensile strength, extensibility, as well as hardness by using GG are the major factors which improved acceptability of noodle product prepared using the traditional method. Gharibzahedi et al. (2017) also confirmed that the development of crystallinity in the structure of the fresh yellow alkaline noodle improved its textural properties such as tensile strength and elasticity.

However, XG significantly reduced some textural parameters because of more solids leaching out during cooking (higher cooking loss) and starch granules collapsing (greater expansion ratio). These results are different from those reported in Kraithong, Lee & Rawdkuen (2018), who found that XG significantly improved the texture properties of the rice noodle that was prepared according to the conventional method. Consequently, the generation of heat and pressure from the extrusion process could reduce the ability to improve noodle textural properties of the hydrocolloid, owing to the interruption of polymer chain interactions (Giles Jr, Wagner & Mount, 2013). In contrast, the greater capacity of XG to absorb water (higher rehydration) could be a cause for higher adhesiveness or stickiness in the extruded noodle. In general, the decreases in tensile strength, extensibility, and hardness but increases in adhesiveness as well as stickiness may contribute to a lower acceptability evaluation for rice noodle product (Kraithong, Lee & Rawdkuen, 2018). Consequently, we found that the lower acceptability score was found in the extruded noodle added by XG. Niu et al. (2017) also confirmed that lower tensile strength and extensibility led to lower acceptance for noodle products.

In fact, hydrocolloids could improve the springiness or elasticity in food texture because of the network development throughout their polymer chains (Suwannaporn & Wiwattanawanich, 2011). Nevertheless, an improvement of properties for extruded noodles was not observed in this study. This could be because the network development of the hydrocolloids could be partially disrupted by pressure and shear force that is generated during the extrusion process (Giles Jr, Wagner & Mount, 2013).

## Conclusions

The uses of GG and CMC excellently improved the quality attributes of rice noodle prepared under the extrusion condition. They significantly improved cooking tolerance and textural properties of the extruded rice noodle. Ultimately, GG 0.2% was the best hydrocolloid for improving rice noodle quality in terms of strengthening noodle structure and improving texture properties. Moreover, this hydrocolloid also gave the most flexible texture for the extruded noodle, indicated by the highest tensile strength and extensibility. These because the use of hydrocolloid could provide the best thermal stability indicated by the lowest cooking loss. Besides, using the gum also caused less disruption of starch granules during the extrusion process, proved by the greater maintained crystallinity and lower expansion ratio. All the improvements frequently contribute to higher acceptability of the product. Unfortunately, XG which is a polysaccharide with a highly branched molecular structure could not be used to improve the qualities of the extruded rice noodle because it gave a weak structure for the noodle. This is because using the XG caused less heat tolerance and unstable structure for the rice noodle, proved by the extensive increase in expansion ratio and cooking loss, as well as a decrease in crystallinity. Evidently, XG was not a proper hydrocolloid to improve the extruded noodle qualities. TPC, antioxidant activities, color, and XRD-pattern of the extruded rice noodle were not affected by using GG, CMC, and XG. Also, no effects of hydrocolloid level on the qualities of the extruded noodle were found.

##  Supplemental Information

10.7717/peerj.10235/supp-1 Supplemental Information 1 Raw data

## Additional Information and Declarations

### Data Availability

The following information was supplied regarding data availability: The data is available in the Supplemental File.
